# Functional human regulatory T cells fail to control autoimmune inflammation due to PKB/c-akt hyperactivation in effector cells

**DOI:** 10.1186/1546-0096-9-S1-O7

**Published:** 2011-09-14

**Authors:** EJ Wehrens, G Mijnheer, B Vastert, M Klein, J van Loosdregt, W de Jager, PJ Coffer, BJ Prakken, F van Wijk

**Affiliations:** 1 Center for Molecular and Cellular Intervention, Wilhelmina Children’s hospital, University Medical Center Utrecht; Lundlaan 6, 3584 EA Utrecht, The Netherlands; 2 Molecular Immunology Lab, Department of Immunology, Wilhelmina Children’s hospital, University Medical Center Utrecht; Lundlaan 6, 3584 EA Utrecht, The Netherlands

## Background

During the last decade research has focused on the application of FOXP3^+^ regulatory T cells (Treg) in the treatment of autoimmune disease. However, thorough functional characterization of these cells in patients with chronic autoimmune disease, especially at the site of inflammation, is still missing.

## Aim

Here we studied Treg function at the site of autoimmune inflammation in patients with juvenile idiopathic arthritis (JIA).

## Methods

Mononuclear cells were isolated from peripheral blood (PB) of both patients and healthy controls (HC) and from synovial fluid of JIA patients. To study Treg function, in vitro suppression assays were performed measuring both T cell proliferation and cytokine production in the culture supernatant. In addition, responsiveness to TGFβ mediated suppression was investigated by culturing effector cells in the presence of TGFβ.

## Results

Treg from both the peripheral blood as well as the inflamed joints of JIA patients were fully functional. Nevertheless, Treg-mediated suppression of cell proliferation and cytokine production by effector cells from the site of inflammation was severely impaired, due to resistance to suppression. In these inflammatory effector cells, activation of protein kinase B (PKB)/c-akt was enhanced and inhibition of this kinase restored responsiveness to suppression.

## Conclusions

PKB/c-akt hyperactivation causes resistance of effector cells to suppression at the site of inflammation in human autoimmune disease. These findings suggest that for a Treg enhancing strategy to be successful in the treatment of autoimmune inflammation, resistance of effector cells to suppression should be targeted as well. This might be achieved by selectively inhibiting PKB/c-akt activation.

